# Stability Depends on Positive Autoregulation in Boolean Gene Regulatory Networks

**DOI:** 10.1371/journal.pcbi.1003916

**Published:** 2014-11-06

**Authors:** Ricardo Pinho, Victor Garcia, Manuel Irimia, Marcus W. Feldman

**Affiliations:** 1 Department of Biology, Stanford University, Stanford, California, United States of America; 2 PhD Program in Computational Biology, Instituto Gulbenkian de Ciência, Oeiras, Portugal; 3 Institute of Integrative Biology, ETH Zurich, Zurich, Switzerland; 4 EMBL/CRG Research Unit in Systems Biology, Centre for Genomic Regulation (CRG), Barcelona, Spain; Pennsylvania State University, United States of America

## Abstract

Network motifs have been identified as building blocks of regulatory networks, including gene regulatory networks (GRNs). The most basic motif, autoregulation, has been associated with bistability (when positive) and with homeostasis and robustness to noise (when negative), but its general importance in network behavior is poorly understood. Moreover, how specific autoregulatory motifs are selected during evolution and how this relates to robustness is largely unknown. Here, we used a class of GRN models, Boolean networks, to investigate the relationship between autoregulation and network stability and robustness under various conditions. We ran evolutionary simulation experiments for different models of selection, including mutation and recombination. Each generation simulated the development of a population of organisms modeled by GRNs. We found that stability and robustness positively correlate with autoregulation; in all investigated scenarios, stable networks had mostly positive autoregulation. Assuming biological networks correspond to stable networks, these results suggest that biological networks should often be dominated by positive autoregulatory loops. This seems to be the case for most studied eukaryotic transcription factor networks, including those in yeast, flies and mammals.

## Author Summary

Multicellular organisms show an incredible diversity of cell types in their different tissues. Functional classes of cells can be attributed to the activation and repression of genes, which enable each cell type to support different functions within the organism. These patterns of activity have been studied by means of gene regulatory networks (GRNs). How these gene networks generate stable phenotypic states is thought to underlie the development and evolution of organisms. The pathways to these states are influenced by the autoregulatory properties of these networks. The stability and robustness of gene networks are used to investigate how such states are maintained. This study sheds light on how these properties relate to one another. By simulating the evolution of these networks, we show that genes depend on positive self-regulation to remain stable and robust when faced with random mutations or environmental perturbations. Assuming biological networks correspond to stable networks, our results suggest that biological networks should often be dominated by positive autoregulatory loops. This seems to be the case for most studied eukaryotic transcription factor networks, including those in yeast, flies and mammals.

## Introduction

Gene regulatory networks (GRNs) are believed to play a central role in organismal development and evolution [1]–[3]. Recent theoretical and experimental studies have revealed that GRNs have many interesting quantitative and qualitative features, including scale-free structure [4], recurring motifs [5], robustness [6], and evolvability [7]. Here we focus on a very specific and common network motif, autoregulation [8], and its contribution to stability and mutational robustness [9].

A direct autoregulation motif in transcriptional GRNs consists of a regulator that binds to the promoter region of its own gene, thus regulating its own transcription. It constitutes the simplest case of a feedback mechanism. Two thirds of *E. coli*'s transcriptional factors (TFs) are believed to be autoregulated [10]. The fraction of autoregulated TFs is lower for yeast (10% [11]), but extensive autoregulation at the post-transcriptional level has been suggested [12]. Two rules relating the presence of feedback loops in GRNs to their dynamical properties have been proposed [13]: (i) a necessary condition for multistability (i.e., the existence of several stable fixed points in the dynamics) is the existence of a positive circuit in the regulatory network (the sign of a circuit being defined as the product of the signs of its edges); and (ii) a necessary condition for the existence of an attractive cycle in the dynamics is the existence of a negative circuit.

These two types of dynamical properties have been associated with important biological phenomena: cell differentiation and stochastic switching in the first case [14], homeostasis [9] and periodic behaviors (e.g., cell cycle [15] and circadian rhythms [16]) in the second. Although these conditions are necessary, they are often not sufficient to define network dynamics, which can depend on other details of the GRN model [13]. For example, negative autoregulation (NAR), the shortest negative circuit possible, has been traditionally associated with robustness of gene expression to noise [9]. However, if the NAR feedback contains a long delay, noise may be amplified [17]. Moreover, both positive and negative feedback circuits are usually embedded in larger networks, and the relative contributions of multiple positive and negative feedback loops to the dynamics of a whole network are largely unknown [13], [14], [18]–[20].

Here, we investigate the relationship between the sign of autoregulation and the stability and mutational robustness of genetic networks. We study this in the context of a widely used gene network model [21]–[23], related to the modeling framework of Boolean networks [24]. We find that stability and robustness are highly correlated with the sign of autoregulation, and that selection for stability leads to positive autoregulation. Despite these positive associations, we show that selection does not maximize robustness and that it is possible to engineer networks with higher robustness by manipulating their diagonal and off-diagonal elements. We also show that autoregulation is conserved over time and that evolved networks are a special subset of stable networks (networks that show fixed point dynamics) with high robustness. Finally, we discuss some implications for biological systems and compare our results with biological networks of different organisms.

## Methods

### Developmental model

To study how stability, robustness and autoregulation change during evolution, we use a standard model for GRN. In one generation, we assume that that the *phenotype* of an organism *S*(*t*) develops over time *t*, starting from an initial phenotype *S*(*t* = 0), under the influence of a gene-interaction network *W*. In general, phenotypes are thought of as expression levels of the genes of the organism at time *t*. Thus, they are vectors of dimension *N*, 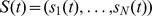, with binary entry values 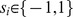, where *N* is the number of genes of the organism.

Phenotypes *S*(*t*) change by the action of a gene-interaction network that drives their *development*, and is represented by an 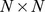 matrix, *W*, whose elements, *w_ij_*, denote the effect on gene *i* of the product of gene *j*. These interaction weights *w_ij_* are nonzero and binary, 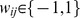. Thus, all genes either repress or activate each other's expression.

In this study, we assume that size of the gene interaction network is *N* = 10 genes. The matrix *W* is not necessarily symmetric. Diagonal elements, *w_ii_*, represent autoregulation, i.e., the action of the *i* ^th^ gene on itself.

Each network *W* determines the dynamics of the phenotype *S*(*t*) in a series of *development steps*. The repeated application of such development steps on a phenotype results in deterministic, discrete-time dynamics of *S*(*t*), modeled by the set of nonlinear coupled difference equations:
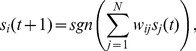
(1)where *sgn*(0) = 1. This spin glass or neural network-type model [22] represents a subclass of Random Boolean Networks [24] known as Random Threshold Networks [25].

When simulating development, the network is updated synchronously, that is, only values of *s_i_* from time step *t* are used for the calculation of *s_i_* (*t*+1) (see [26]–[28] for asynchronous updates.) We refer to Equation (1) as the *development process* (see [23], [29] for model illustration, biological motivation and assumptions).

The development process can be extended to include *sparse networks G*. Sparse networks are used to model gene interactions in which only a fraction of the genes repress or activate a fraction of all the other genes, in contrast to fully connected networks W, where all genes have some effect on all other genes.

Let *G* denote an interaction network represented by a 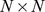, *N* = 10 square matrix whose entries, *g_ij_*, take the values of {−1,0,1}. The parameter *c*, the *density* of the network, determines the proportion of non-zero matrix elements. When simulating sparse networks (see Results), we chose *c* = 0.2 (due to similarity to the biological networks in Table 1) and a regular, directed graph topology, where all genes have degree 2. This means that all genes in a network are regulated by two genes and also regulate two other genes.

**Table 1:** Most studied eukaryotic transcription factor networks (including yeast, flies and mammals) show values of *p* ranging from 0.76 to 1.

Species	TF System	*p*	# autoregulated	# TFs	Reference
Mammals	core pluripotency network	1	5	5	[42]
Drosophila	gap genes	1	4	4–7	[48], [49]
Drosophila	segment polarity	1	5	10	[37]
Drosophila	circadian clock	1	5	6	[50]
Arabidopsis	circadian clock	1	4	4	[51]
Arabidopsis	flower morphogenesis	1	2	10	[38]
Mouse	blood stem cells	1	7	11	[52]
Human	genome-wide	0.76	21	301	[46]
Drosophila melanogaster	genome-wide	0.78	14	87	[46]
Saccharomyces cerevisiae	genome-wide	0.75	12	169	[46]
E. coli	genome-wide	0.26	109	182	[45]
Sea urchin	endomesoderm development	0.25	16	50	[53]

Description of different regulatory networks of different organisms in terms of size, ubiquity and sign of autoregulation.

#### Examining model behavior

Starting from an initial gene expression state the system described in Equation (1) will eventually reach an *attractor*. Such an attractor may be a fixed point or a limit cycle. In a biological context, a fixed point can be interpreted as one mature phenotype of the organism after the completion of development.

#### Simulation experiment setups

To investigate how specific network features change within populations under development as well as under evolution, we devised two main simulation experiment setups.

Each organism is represented by a network *W* and an initial state *S*(0). In this study we limit ourselves to two experimental setups: pairs of randomly chosen networks and random initial conditions (*RNRC setup*), and *n* randomly chosen networks that each act on one single randomly chosen initial condition (termed *RNIC setup*). We use RNRC for populations that don't evolve, and RNIC for populations subject to evolution (as explained below). The *random initial conditions* for the simulation experiments are generated by sampling one phenotype with uniform probability from the entire phenotype space (*s_i_*(0) = 1,−1 with probability 0.5).

### Random and stable networks

To generate a *random* network *W_r_* the matrix-elements *w_ij_* are sampled from {−1,1} with equal probability (0.5 per element and entry). Additionally, we can generate *stable networks W_s_* with a pre-selection procedure. In this procedure, a random network W_r_ and random initial state pair are first generated. This pair undergoes the development process. If no fixed point is attained, a new pair is sampled and developed. This step is repeated until some (*W*, *S*(0))- pair generates a fixed point. The final network *W_s_*, is a stable network. This notion of stability refers to an *individual level stability*, which differs from a notion of *population level stability* that will be introduced below.

In this study, we refer to stability as the property of a network, while strictly speaking, it is a property of a *W*, *S*(0) pair. However, we have previously shown [30] that the network is by far the most important determinant of stability. If a network is stable/unstable with a random initial state, it most likely remains stable/unstable with any other initial state. For this reason, we classify networks as stable or unstable, even if we just solve Equation (1) for one possible initial state.

### Evolved and non-evolved networks

Two types of simulation experiments are our primary focus in this study. First, experiments in which a population of organisms undergoes the development process only, which we refer to as *non-evolved*. Secondly, experiments with multiple generations (*evolved*), where after each development process (one generation) the composition of the population of organisms is additionally altered by evolutionary mechanisms. The development process is completed after all organisms have reached some stage of development: either a fixed point or a cycle. We implemented standard evolutionary mechanisms, such as selection, mutation and recombination. After these evolutionary forces have acted on the population, a new development process starts in the next generation with identical initial phenotypes for each organism.

In this study, we set the population size to *n* = 500 across all experiments (unless otherwise noted). This population size remains constant during evolutionary simulations (Wright-Fisher model with sampling with replacement).

### Selection

To study how selection affects evolving populations, we implemented different *types of selection* or *selection models*. The mutation and recombination mechanisms applied were the same for all evolved populations.

Selection mechanisms modify the number of copies of one specific network within the population depending on the fitness of the phenotype that specific network has generated through development. In a selection mechanism, one phenotypic state can be marked as the optimal state, with the highest possible fitness. If such an optimal state, *S* ^opt^(∞) is specified, the fitness of a network with attractor *S*(∞) is given by:
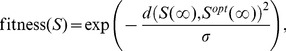
(2)where *d* is the normalized Hamming distance and *σ*>0 determines selection strength [23].

Small values of *σ* imply strong selection against deviations from the optimal state. Large values minimize the fitness difference between phenotypes. The Hamming distance *d* corresponds to the number of differing expression states of individual genes between two phenotypic states [31], subsequently normalized to the interval [0,1] in this study.

Equation (2) is valid under the assumption that *S* ^opt^(∞) and *S*(∞) are attractors with identical, optimal cycle lengths *l*_opt_. The cycle length of the attractor with highest fitness is denoted with *l*_opt_. The fitness of attractors of length *l*≠*l*_opt_ depends on the selection model. We use attractor length and cycle size or period as synonyms.

### Selection models

We implemented selection models similar to those used by other authors [23], [29] and also introduced new ones. In these models, the fitness of a developed organism depends on two parameters: selection strength, *σ*, and optimal period, *l*_opt_.

**Selection model 1 (selecting for stability): *l*_opt_ = 1, fitness(*l*≠*l*_opt_) = 0**

***σ*** **= 0.1 ‘target’ model**

 Selects for fixed points and an optimal gene expression state. Fitness is given by Equation (2) for fixed points and is 0 for cycles.

***σ*** **= ∞ ‘no target’ model**

 Fitness is 1 for all fixed points and 0 for cycles.

**Selection model 2 (selecting against stability):** ***l*** **_opt_>1, fitness(** ***l*** **≠** ***l*** **_opt_) = 0**

***σ*** **= ∞,** ***l*** **_opt_ = 2,3,…,7 (cycles)**

 We generalize the ‘no target’ model to select for cycles. Fitness is 1 for cycles of length *l* = *l*_opt_ and 0 otherwise, including fixed points. We try different *l*_opt_>1.

**Selection model 3 (neutral for stability):**

***σ*** **= 0.1, fitness = max(fitness(** ***S*** **)) for all** ***S*** **in** ***S*** **(∞), any** ***l*** (S represents any state in the attractor S(∞))

 We generalize the ‘target’ model to not require stability. When *l* = 1, we have the ‘target’ model as a special case and fitness is given by Equation (2). When *l*>1, fitness is the maximum fitness given by Equation (2) for all states in the cycle. The attractor *S*(∞) can be a fixed point or a cycle.

**Selection model 4 (random sampling):**

***σ*** **= ∞, fitness = 1, any** ***l***

 No selection. We take this as the null model.

 For each selection model, we generated between *z* = 100 and *z* = 300 independent populations (depending on the model). Specifically: *z* = 200 for the ‘target’ model; *z* = 300 for the ‘no target’ model; *z* = 200 for Selection model 2; *z* = 100 for Selection models 3 and 4. We denote such an aggregation of populations as a *set of populations* and *z* as its *set size*. Each evolved population has a different initial state, but all individuals within the same population have the same initial state.

### Mutation

Mutations randomly change the sign of *w_ij_* at a rate *μ* = 0.1 per network per generation. All matrix entries, *w_ij_*, including diagonal elements, *w_ii_*, have equal probability of changing sign, namely *μ*/*N* ^2^ = 0.001 per generation. For sparse networks we use a probability for changing sign of *μ*/(*c N* ^2^).

### Recombination

To model recombination we follow the methods in [23], where full chromosome segregation (no crossover) is implemented. The two offspring of a randomly chosen pair of recombinant parents are generated by randomly taking half the rows from each parent matrix. This procedure is performed on the entire population.

### Population metrics

We define a population-level *stability* (henceforth referred to as *stability* if not otherwise stated) as the fraction of networks that are stable (individual-level) in a given population [30]:
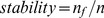
(3)where *n_f_*≤*n* is the number of times the attractor is a fixed point, and *n* is population size, that is, the number of network matrices. Stability takes values between 0 and 1.

Similarly, we define the *robustness* of a population as the fraction of all possible mutated networks in a population that reach the same fixed point attractor as their un-mutated originals [23], conditional on the fact that the attractor did not become a limit-cycle. Specifically, we estimate robustness by looping through the population of networks and mutating every element of each network matrix W (changing the sign of *w_ij_* for binary matrices), thus generating *N^2^* single-mutants per network. Then, the 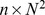 networks undergo the development process starting from the same initial phenotypes as their originals, and are further analyzed.

For a single network, we define individual-level viability as the fraction of single-mutants that attain a fixed point:
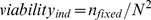
(4)where *n_fixed_*<*N* ^2^ is the number of times the *N* ^2^ single-mutants have still generated a fixed point. With this metric, we can now define individual-level robustness:
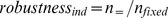
(5)and *n*_ = _≤*n_fixed_* is the number of times the same attractor state as the one attained by the un-mutated original is reached starting with identical initial conditions (i.e., the mutant has the same phenotype as its wildtype). The *population-level viability* and *robustness* measures are computed from the averages of all networks in the entire set of populations.

Both robustness and viability take values between 0 and 1. In normalizing robustness by *n_fixed_* instead of *N* ^2^, we attempt to decouple the effects of stability and robustness. In the vast majority of cases, mutations that change the stability of a network do not affect its robustness score. Exceptions to this are the rare occasions when *n_fixed_* = 0 (robustness is not defined), or when *n_fixed_* is low (robustness can only take a few specific values).

In an extension of the fraction of activating connections-statistic [32], we found it useful to measure properties of diagonal and off-diagonal elements of a matrix *W* separately, thereby decoupling the effects of direct autoregulation and off-diagonal regulation. For a single network matrix *W*, we define:
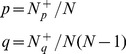
(6)where *N^+^_p_* and *N^+^_q_* are the number of positive diagonal and off-diagonal elements of *W*, respectively. Both *p* and *q* are always positive and take values between 0 and 1. We call *p* the *sign of autoregulation*, because autoregulation is predominantly positive when *p*>0.5 (we call this *positive autoregulation*), and mostly negative when *p*<0.5 (we call this *negative autoregulation*).

The metrics *p* and *q* measure direct regulatory influence. However, network dynamics can also be affected by long-range interactions. To assess the role of such long-range regulation, we introduce a metric of *indirect positive autoregulation r*, which measures the fraction of autoregulatory paths over two genes that are positive (i.e., gene A activates gene B, which activates gene A). For a single network *W*, we define:
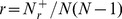
(7)where *N^+^_r_* is the number of positive off-diagonal elements of *WW^T^* (*W^T^* is the transpose of W). Because *WW^T^* is symmetrical, it suffices to count the fraction of positive entries in either of the triangles of the matrix.

We also define metrics to assess the population-average of gene interaction strengths:
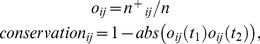
(8)where *n^+^_ij_* is the number of positive elements in position *i*,*j* across all *n* networks in a population, *abs* is the absolute value function, and *t*_1_ and *t*_2_ are two different evolutionary time points. Here, *o_ij_*, is referred to as *average positive ij- interaction strength*, and measures how much gene *i* activates gene *j* on average, whereas the conservation statistics between population-averaged gene interaction strengths measures how much these interaction strengths are maintained over time. In evolutionary experiments, conservation, *p*, *q*, and *r* are averaged over all individuals and all populations.

The code utilized in this paper can be downloaded from https://github.com/rpinho/phd.

## Results

### Individual- and population-level stability and autoregulation are correlated in Boolean GRNs

To study the relationship between the sign of autoregulation (*p*) and stability during development, we devised two experiments. First, for each *p* = 0, 0.1, …, 1, a pair consisting of one random network and one random initial condition was sampled (RNRC setup; see Methods). Equation (1) was then evaluated for each pair: if the attractor was a fixed point, the network was considered stable. Instead, if the solution to Equation (1) was a limit cycle, the network was considered unstable. This process was repeated *n* = 10^5^ times for each *p*.

**Figure 1:**
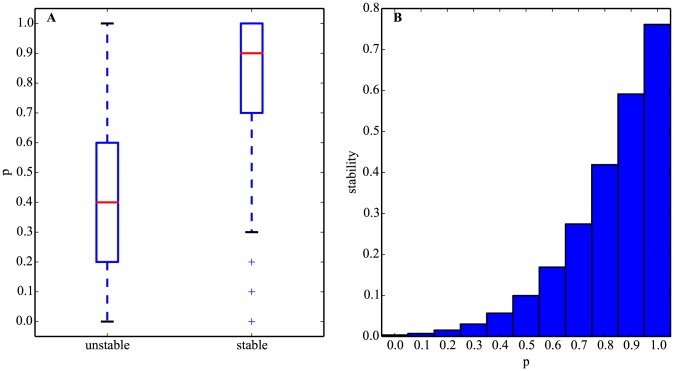
Positive autoregulation favors stability. A. Boxplots of the sign of autoregulation (*p* in Equation (6)) for two network classes: stable (fixed points) and unstable (cycles). Whiskers are 1.5 times the inter-quartile range (the difference between the first and third quartiles). All outliers are represented. B. Histogram of the values of *p* for stable matrices, i.e., stability (Equation (3)) as a function of *p*. The dotted line is an exponential function of *p*. Networks are random and non-evolved. Panels A and B represent the same data in different form. Equation (1) is solved *n* = 10^5^ times for each *p* = 0, 0.1,…, 1 (11 bins).

Figure 1A shows that individual-level stability is strongly associated with *p*. Stable networks have significantly higher values of *p* than unstable networks (median of 0.9 compared to 0.4 for unstable networks; Mann-Whitney U p-value ∼0, Figure 1A).

This positive association was also observed for population-level stability in a second experiment. We subdivided the networks generated in the first experiment into populations of identical *p*, and measured the average *population-level stability* for each *p*. We observed that the fraction of stable networks increases rapidly with higher values of *p* (Figure 1B). These results also indicate that *p* and stability are strongly associated.

### Evolution of autoregulation when selecting for stability is non-linear

We next studied how *p* changes when explicitly selecting for and against individual-level stability in evolutionary simulations. To this end, we founded sets of populations with random networks and the same initial state for each population (RNIC setup; see Methods) [23], [29]. The average *p* was set to *p* = 0.5 at generation 0. We then evolved all populations under the six different selection models (including mutation and recombination) described in the Methods. For all of these selection models, we followed the evolution of the sign of autoregulation *p* over 10^6^–10^7^ generations (until equilibrium was attained).

**Figure 2:**
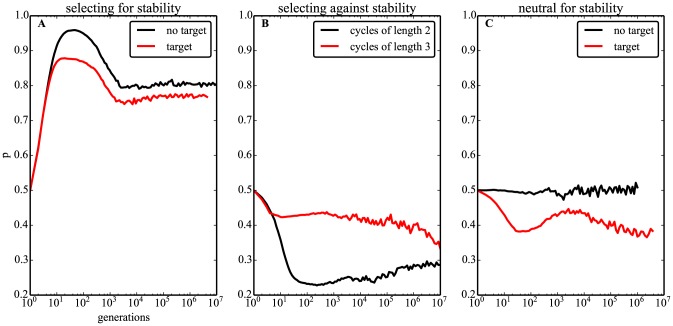
Stability selects for positive autoregulation. Time evolution of *p* (Equation (6)) on the vertical axis for different selection models. A. Selecting for stability: only fixed points are allowed to survive. ‘no target’ means all fixed points have fitness of 1 and all cycles have fitness of 0. ‘target’ means selection for a specific fixed point, with fitness given by Equation (2) as a function of the hamming distance from the target phenotype. B. Selecting against stability: only cycles of specific length are allowed to survive. Shown are selection for cycles of size 2 and 3. C. Neutral for stability: fitness does not depend on stability. ‘no target’ means random sampling. ‘target’ means selection for a specific fixed point, with fitness given by the maximum value of Equation (2) applied to all points of the attractor as explained in Methods (allows cycles of all sizes including fixed points). Sample size is *n*∼10^5^ evolved networks per generation.

Consistent with the observations for non-evolving networks, positive autoregulation is strongly favored during evolution, both under the ‘target’ and ‘no target’ models (Figure 2A). However, the evolution of *p* follows a complex, non-linear pattern. After a sharp initial increase over the first ∼50 generations, *p* reaches its maximum when population-level stability is above 95% (stability-metric not shown in the Figure), and starts to decay slowly to a stable evolutionary equilibrium of *p*∼0.8 from *t*∼10^3^ generations for both models (Figure 2A). At the peak, a fraction of up to *p*∼0.95, or 19/20 surviving matrices show positive autoregulation for all genes under the ‘no target’ selection model.

Interestingly, selecting for cycles of length *l* = 2 (i.e. against individual-level stability), has the opposite effect on evolving networks: *p* decreases sharply down to ∼0.3 (Figure 2B), leading to negative autoregulation. A similar, but less pronounced pattern is observed when selecting for longer cycles with lengths *l*>2 (Figures 2B and S2).

As expected, a neutral model with no selection for individual-level stability or a specific target produces random networks, with values of *p* centered on *p*∼0.5 (Figure 2C). However, mean values of *p*<0.5 also evolve when not selecting for any particular attractor length, but still selecting for a specific target (Figure 2C).

Thus, the selection for stability leads to positive autoregulation.

### Autoregulatory motifs are highly conserved over time

These results suggest that selection may act over direct autoregulatory motifs (i.e. the diagonal elements of the GRN matrix) to promote individual-level stability. If this is the case, positive diagonal elements should be overrepresented across evolved populations relative to off-diagonal elements, since selection for individual-level stability could be achieved by maximizing autoregulation *p*.

To test this hypothesis, we calculated the average interaction weights 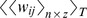 in populations evolved from a RNIC setup (the number of populations, *z*, was 100, and the population size *n* = 500). The metric 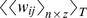 is the average value of the *w_ij_* entry of individual networks *W*, taken across a set of populations (including individuals within the populations) as well as evolutionary time. Extreme values of 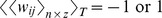 indicate that the matrix element *i*,*j* is identical across individuals, different populations and different generations, whereas a neutral value of 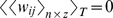 means the matrix element *i*,*j* fluctuates randomly in individuals and populations and is not conserved over time.

We found that, in our evolution experiments, the averaged diagonal elements 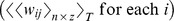 attained higher values than the off-diagonal elements, consistent with stronger selection for positive autoregulation acting on the diagonal elements (Figure S3). This is further supported by the observation that selecting for individual-level stability leads to positive values of 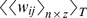, whereas selecting against individual-level stability leads to negative 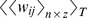 values. Selecting neither for nor against stability, but still selecting for a specific target, also yields negative 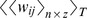.

To increase our confidence that the value of *p* = 0.8 emerging under the no-target model is maintained by selection for stability, we compared the effects of diagonal and off-diagonal mutations on individual-level stability in the evolved networks. We hypothesized that since single mutations in diagonal elements could result in unstable networks more often than in off-diagonal elements, networks carrying diagonal entry mutations would therefore be weeded out at higher rates. Because p and stability are correlated, p would then be maintained at a high value.

**Figure 3:**
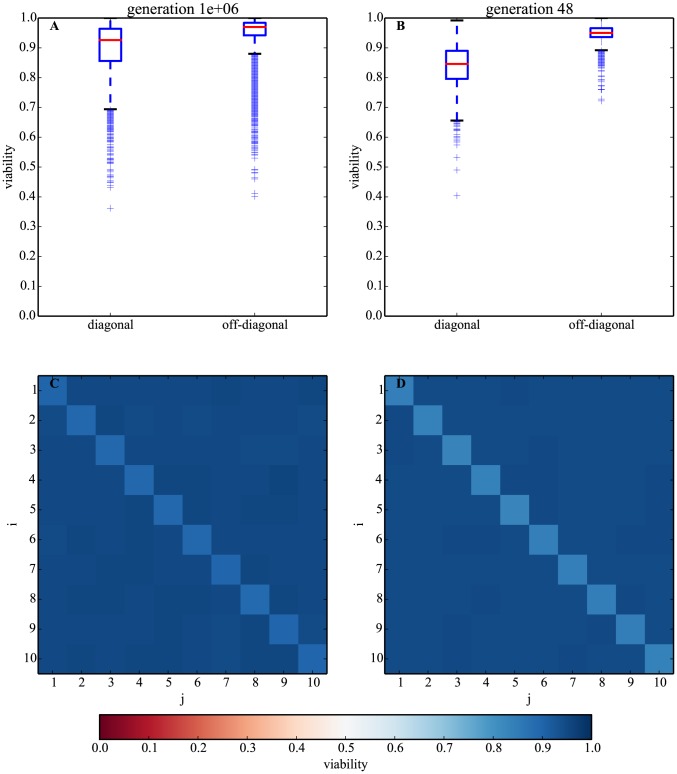
Changing the sign of an autoregulatory element is more likely to kill a network than a random mutation. A. Boxplots of average viability (Equation (4)) for diagonal and off-diagonal elements of the network matrices at evolutionary equilibrium (*t* = 10^6^ generations). B. Same as (A) but at *t* = 48, when *p* (Equation (6)) is largest. C. Heat map of average viability for all matrix elements at *t* = 10^6^ generations. Darker blue (off-diagonal elements) means higher viability after mutation than lighter blue (diagonal elements). D. Same as (C) but at *t* = 48. Sample size is *n*∼10^5^ evolved networks per generation.

In a first approach, we sampled *n* = 10^5^ stable networks at *t* = 10^6^ generations (equilibrium reached) evolved under the ‘no-target’ model. The overall fraction of networks that survive after acquiring single mutations (viability in Equation (4)) is high (95%). Consistent with our hypothesized maintenance mechanism, the population-level viability of networks is significantly higher for off-diagonal elements (median of 0.97 compared to 0.93 for diagonal; Mann-Whitney U p-value∼0, Figure 3A). Intriguingly, the difference in viability is even higher for *t* = 48 generations, when *p* is close to maximum (Figure 3B), and the evolutionary dynamic has not yet reached equilibrium. These results suggest that a mutation of a diagonal element is more likely to lead to a cycle than mutation of off-diagonal elements.

In a second approach, we studied the conservation of values in diagonal versus non-diagonal elements between two given time points. To this end, we sampled *n* = 10^5^ evolved stable networks at *t*_1_ = 10^5^ and *t*_2_ = 10^6^ generations from the ‘no-target’ and the random models. Subsequently, we counted the fraction of positive elements *o_ij_*, (Equation (8)) across all networks and for each position in the gene regulatory matrix (*w_ij_*) for both *t*_1_ and *t*_2_, and estimated conservation values as in Equation (8).

**Figure 4:**
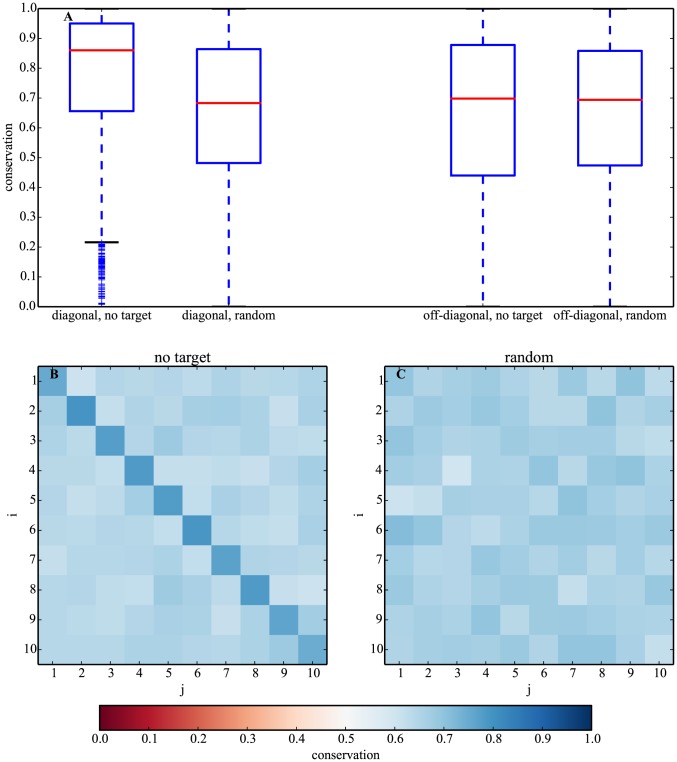
Autoregulation is conserved over time. A. Boxplots of average conservation (Equation (8), *t*_1_ = 10^5^ and *t*_2_ = 10^6^ generations) for diagonal and off-diagonal elements of random and evolved ‘no target’ networks. B. Heat map of average conservation for all matrix elements under the ‘no-target’ model. Darker blue (diagonal elements) means higher conservation than lighter blue (off-diagonal elements). C. Same as (B) but for random networks. Sample size is *n*∼10^5^ evolved networks per generation.

For the no-target model, we found that diagonal entries *w_ii_* with positive sign are significantly more conserved over time than off-diagonal matrix elements (medians of 0.86 and 0.70, respectively; Mann Whitney U p-value∼0, Figure 4A). These diagonal elements under the no-target model are also more conserved when compared to diagonal elements evolved under the random model (medians of 0.86 and 0.68 respectively; Mann-Whitney U p-value∼0, Figure 4A). We found no significant differences in conservation of the off-diagonal entries between the ‘no-target’ and the random models (one-sided Mann-Whitney U p-value∼0.1, Figure 4A). This finding provides further evidence that positive autoregulation is maintained by selection for stability.

### Stability, robustness and autoregulation coevolve

The time course of *p* displays an intriguing complexity. After the stability metric reaches its maximum and ceases to change, *p* keeps evolving and decreases to a lower equilibrium value (Figure 2A). To investigate this behavior, we asked which other network parameters may also affect the evolution of *p*. Since Wagner [23] has previously shown that during network evolution robustness is also (indirectly) selected for when selecting for stability, we studied how this robustness compares with *p* over the course of evolution.

**Figure 5:**
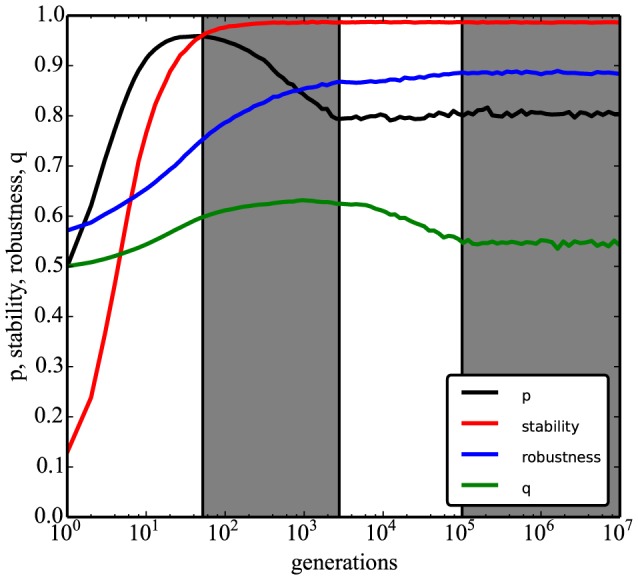
Positive/negative regulation evolves non-linearly with robustness. Coevolution of stability (Equation (3)), robustness (Equation (5)), *p* and *q* (Equation (6)) under selection for stability (‘no-target’ model). Black line (*p*) is the same as in Figure 2A. White and grey regions serve as guides to the eye signaling different phases of evolution. From *t* = 1 to *t*∼48, *p* reaches its maximum and stability reaches 0.96. From *t*∼48 to *t*∼10^3^, *p* approaches equilibrium and *q* reaches its maximum. From *t*∼10^3^ to *t*∼10^5^, robustness and *q* approach equilibrium. From t∼10^5^ onwards, all represented quantities are at evolutionary equilibrium. Sample size is *n*∼10^5^ evolved networks per generation.

Intriguingly, during the simulation experiments under the no-target model, we observed that robustness (Equation (5)) increases with time and appears to coevolve with *p*, reaching its maximum at the same time point at which *p* reaches equilibrium (Figure 5). This association, however, is also not linear: shortly after stability has reached equilibrium, robustness still increases despite the fact that *p* has started decreasing.

### Robustness and autoregulation are associated, but this relationship is dynamic during evolution

Because robustness and *p* reach equilibrium at about the same time, we hypothesized that *p* could be adapting under indirect selection for robustness. In that case, the equilibrium value of *p*∼0.8 would favor higher robustness (or perhaps: “maximize robustness”).

To test this, we generated groups of 10^5^ random stable networks for *p* = {0.1,0.2,0.3,…,1}, and calculated the robustness for each group. Robustness was assessed after running one single development process for each network in the RNRC setup. Only stable networks were considered for this analysis.

**Figure 6:**
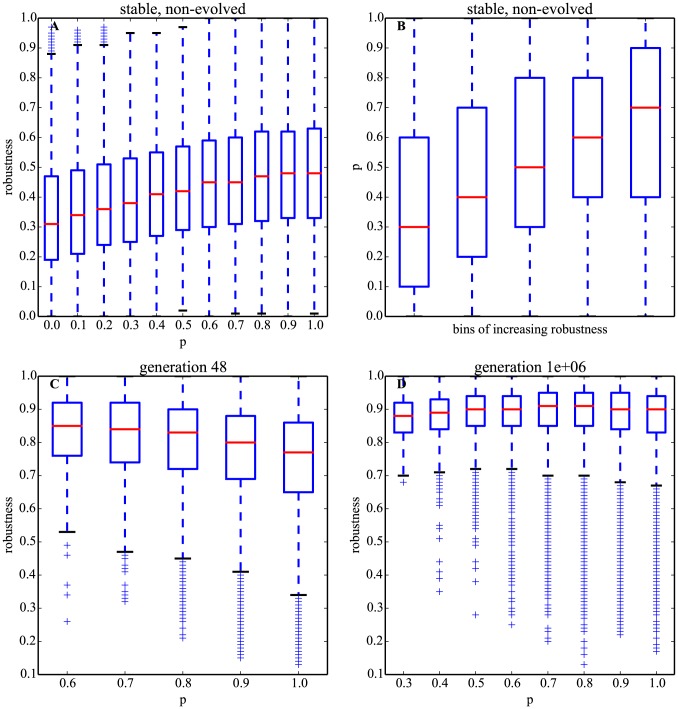
Maximization of robustness during evolution determines the equilibrium values of *p*. The relationship between robustness and autoregulation changes during evolution. A. Boxplots of robustness (Equation (5)) for each *p* = 0, 0.1,…, 1 (11 bins) for stable, non-evolved networks (*q* = 0.5). B. The inverse of (A), i.e., boxplots of *p* (Equation (6)) for 5 bins of increasing robustness, each of size 0.2, from 0 to 1. C. Same as (A) but for evolved networks, at *t* = 48, when *p* is largest. At this stage of evolution, there are no significant samples of networks with *p*<0.6. D. Same as (C) but at evolutionary equilibrium (*t* = 10^6^ generations); there are no significant samples of evolved networks with *p*<0.3. *q* = 0.5 (Equation (6)) for panels A and B but *q* is not constant or controlled for in panels C and D. (It varies with *p* in a non-linear fashion in evolved networks.) Sample size is *n* = 10^4^ networks for each *p* (total n∼10^5^) for (A) and (B). Sample size of evolved networks varies with *p* but total size is *n*∼10^5^ for (C) and (D). Robustness is measured by Equation (5) with *N* ^2^ = 100 mutations per network.

Surprisingly, we found that, similarly to stability, robustness is also positively associated with *p* and does not have a maximum at an intermediate *p*∼0.8 (Figure 6A). Contrary to our hypothesis, robustness of stable, non-evolved networks is maximized by *p* = 1 (see Figure S4 for random networks not pre-selected for stability). This positive association was also observed when representing the data differently: stable networks binned by increasing average values of robustness also show increasing *p* (Figure 6B).

This general positive association is inconsistent with the hypothesized relationship between *p* and robustness after stability reaches its maximum. However, a more in-depth analysis of robustness of *evolving* networks at different time points reveals a completely different picture to the situation in non-evolved matrices (Figures 6C and D).

At both early (*t* = 48, *p*_max_) and late (*t* = 10^6^, *p*_equilibrium_) evolutionary stages, robustness is maximal in evolving matrices with values of *p*<1. At early time points, when *p* has reached its maximum, the relationship between *p* and robustness is fully inverted compared to non-evolved networks, with lower *p* having significantly higher robustness, thus suggesting strong selection for lower *p* to increase robustness (Figure 6C). Strikingly, at equilibrium values, robustness is non-monotonic in *p* and is maximal for *p*∼0.7–0.8, coinciding with the equilibrium value of *p* (Figure 6D). Therefore, these results strongly suggest that, as we hypothesized, it is the maximization of robustness during evolution that determines the equilibrium value of *p*.

### Evolved networks are a distinct subset of stable networks

The above results may seem contradictory: whereas *p* and robustness show a positive association in stable networks generated completely at random (i.e. for all non-evolved stable networks), this association is non-monotonic for stable networks selected by evolution (i.e. evolved networks), where robustness is maximized for more intermediate values of *p* (0.7–0.8). The solution to this apparent paradox might lie in the fact that evolved networks constitute only a subset of all stable networks.

Remarkably, the average robustness for the subset of evolved networks is twice as high as that of non-evolved networks with similar *p* (compare Figures 6A and 6D), suggesting that the relationship between *p* and robustness is modulated by other matrix characteristics on which selection can act.

In order to investigate this possibility, we studied the evolution of the sign of off-diagonal elements (*q*). There are more off-diagonal than diagonal elements; thus the former offer many more targets for mutation. However, a mutation in the off-diagonal has a smaller effect on *q* than a mutation on the diagonal has on *p*. For this reason, q may seem to evolve at lower rates than *p*. More importantly, off-diagonal elements represent regulation of other genes and can form larger and more complex motifs than autoregulatory loops of size one. For this reason, they are harder to study and to interpret. However, under a random model, it is clear that the expected average value of *q* equals 0.5.

The evolution of *q* occurs over a much smaller range than that of *p*, with values spanning from 0.5 to ∼0.63. However, it also shows a non-linear pattern of co-evolution with the other variables (Figure 5). *q* increases up to its maximum approximately until *p* stabilizes, and then it starts to slowly decrease to its equilibrium value at *t*∼10^5^. It reaches equilibrium around the same time as robustness.

These co-evolutionary dynamics suggests that stability and robustness may not only depend on *p*, but also on *q*. Therefore, although robustness is maximized by high *p* for stable networks with *q* = 0.5, the same is not necessarily true when *q*>0.5. This is the case at *q*>0.65, for which robustness is higher for *p* = 0.7–0.8 than for *p* = 1 (Figure S5). Interestingly, these values correspond closely to (*p* = 0.8, *q* = 0.63) of evolved networks at generation ∼1000, when both stability and *p* reach their equilibrium values.

### Engineering super-robust networks: robustness is not fully maximized in evolution

A prediction from our results is that certain combinations of *p* and *q* are more likely to provide stable networks.

To test this, we combined the off-diagonal elements (determining *q*) of stable networks with low *p* and a *q* similar to that of equilibrium (*q* = 0.53 or 0.54), with diagonal elements of high *p* (*p* = 0.9 or 1.0). We call these networks “engineered”. As before, we generated groups of 10^5^ random stable networks for different values of (*p*,*q*) and calculated the robustness for each group. Robustness was assessed after running one single development process for each network in the RNRC setup. Only stable networks were considered for this analysis.

The engineered networks resulted in extremely stable and robust networks (Figure S6A); importantly, randomly sampled stable networks with the same average *p* and *q* are not nearly as stable and robust to mutations (Figure S6B). These observations support the idea that features in the topology of off-diagonal elements of these matrices (i.e., how genes regulate one another) may buffer the destabilizing effects of mutations.

These findings also show that it is possible to engineer networks more robust than those evolved under selection for stability. Thus, robustness is not fully maximized during evolution. In fact, when the founding populations (*t* = 0) have a mean of *q* = 0.9 (rather than being normally distributed around 0.5, see Methods), robustness decreases throughout evolution (Figure S7).

### Positive autoregulatory motifs of length two are also associated with stability and robustness

Direct regulatory influence of genes on one another can explain qualitatively why *p* and *q* are being maximized at the beginning of evolution experiments. Their subsequent decline below the maximum values is related to constraints imposed by the indirect selection on robustness. To further elucidate how these constraints operate, we investigated how long-range interactions embedded in the matrix of direct influences (direct interaction, i.e., *W*) of an organism could contribute to the settling of *p* and *q* below their maximum values.

To this end, we repeated the evolutionary experiments tracking the measure for length-2 autoregulatory interaction *r*, which measures the frequency of networks that contain self-reinforcing interaction loops (gene A activates gene B, which reactivates A, see Methods). We found that *r* is maximized early and attains values above 0.5 throughout evolution, indicating positive long-range autoregulation (Figure S8). Additionally, r lags behind the evolution of *p* and *q*, adapting to selective pressures at lower rates.

To understand why engineered networks are more robust to mutations than random stable networks with the same values of *p* and *q*, we measured *r* for networks similar to the ones shown in Figure S6. We find that *r* is larger for the engineered networks (Figure S9), which explains why engineered networks are more robust for the same values of *p* and *q*.

## Discussion

In this study, we have shown that stability and robustness positively correlate with autoregulation in a Boolean network model of gene regulation, where stable networks have mostly positive autoregulation (*p*>0.5). During evolution in the no-target model, selecting for stability leads to indirect selection for robustness. Strong selection for stability is expressed in the adaptation of direct autoregulatory network properties summarized by *p*, which is maximized early in evolution. The subsequent decline of *p* is explained by additional autoregulatory effects stemming from long-range gene interactions that allow maintenance of high stability values, while simultaneously increasing robustness.

We have limited this study to small networks of 10 genes, comparable to some sub-circuits in genomes found in organisms, summarized in Table 1. We hypothesize that larger gene numbers would lead to similar results. In previous work we have shown that stability decreases with network size, which we simulated for up to *N* = 10^4^ for sparse networks (*c* = 0.2) with scale-free topology [30].

We expect such a decrease in stability with *N* to increase the direct selective pressure on stability, as well as the indirect selective pressure on robustness in an evolutionary experiment. This is supported by the finding that large networks show a increase in robustness after selection for a target compared to small networks [23].

We have also neglected the role of bistability in the evolution of the networks. In other models of gene-regulatory networks it has been shown that mutational robustness correlates with the robustness of phenotypes to changes in initial conditions of the networks *R*_i_ [33]. If a similar correlation exists for the model presented in this study, we would expect indirect selection for less multi-stable networks due to the indirect selection for higher *R*_i_. Networks with high *R*_i_ are expected to have large basins of attraction, decreasing the number of possible fixed points and thus multi-stability.

The model presented here deviates in some important aspects from Wagner's model [23] and Siegal & Bergman's model [29] by which it is inspired. In particular, our model only includes binary matrix elements, whereas [23], [29] allow for real valued entries. Also, in contrast to [29], the normalization function used is not a sigmoidal, but a sign function. That results in the state vectors having real values in [29], whereas in our model we only allow for binary states.

The motivation to deviate from [23] lies in the focus on the sign of autoregulation. Since previous work [30] has shown that the behaviors of real-valued and binary-valued networks show little or no qualitative difference in the context of the questions asked here, it is technically more feasible to implement the easier, binary form of networks. Furthermore, most of the knowledge about gene regulatory networks exists in binary form, given as qualitative information about activation or repression interactions between genes. Thus, to make comparisons with the available data calculated on the basis of binary data, it was justified to limit the study to binary networks.

The assumption that a population of organisms consists of random networks or has random initial conditions is unrealistic. We use random samples of networks or initial phenotypic states because we are interested in the general, overall behavior of populations with respect to some metrics. Random sampling allows us to obtain an unbiased sample of all possible networks, and to capture a part of the heterogeneity in their behavior. To satisfy more realistic assumptions, a sub-space of phenotypes that corresponds to more realistic biological phenotypes needs to be specified. How to achieve this is currently unknown, and such a restriction would have amounted to studying random initial conditions.

The Boolean network model of gene regulation has recently been shown to predict specific patterns of protein and gene activity observed in a wide diversity of biological systems, including yeast [34], [35] and mammalian [36] cell cycles, embryonic segmentation in D. melanogaster [36], [37], and flower development in A. thaliana [38]–[40].

Assuming biological networks correspond to stable networks [23], [29], [34], our results suggest that biological networks should often be dominated by positive autoregulatory loops (i.e. have high *p*). This seems to be the case for most eukaryotic transcription factor networks (including yeast, flies and mammals), with various studies showing values of *p* ranging from 0.76 to 1 (Table 1; with the exception of early sea urchin developmental gene regulatory networks), and with autoregulatory loops being highly conserved across vertebrates [41].

Moreover, in some cases, the presence of strong positive autoregulatory loops seems to be crucial to achieving a stable biological state. For example, in mammalian embryonic stem cells, the core pluripotency network of Oct4, Sox2 and Nanog (plus Klf4 and Esrrb [42]) forms a tight autoregulated circuit, in which each gene activates its own expression as well as the expression of the others, and these interactions are crucial to maintaining a stable pluripotent state [43]. Furthermore, this autoregulatory circuit is likely behind the capacity of somatic cells to be reprogrammed into induced pluripotent stem (iPS) cells when reprogramming factors are expressed exogenously [44].

On the other hand, negative autoregulation seems to dominate in the bacterium E. coli (*p* = 0.26) [45]. Stewart and coworkers [46] have recently suggested that this difference may be due to the presence/absence of sexual reproduction. To test this hypothesis, we reproduced our simulations for evolution without recombination (see Methods) under the no-target model, as a proxy for a model with asexual reproduction, but obtained essentially the same equilibrium values of *p*, despite divergent intermediate evolutionary dynamics and robustness at equilibrium (Figure S10).

Another caveat may lie in the density of the networks employed in our simulations. Biological networks are often sparse [47], and may vary between species as well as for different gene regulatory subcircuits within species; however, we have used fully connected networks in our analyses. Thus, we tested Boolean networks with the same average connectivity as some biological networks (average degree of 2, [47]) (see Methods, Sparse Networks). The evolutionary simulations were conducted under the *RNIC setup* (no pre-selected networks, see Methods), in a similar fashion to the previous simulations. We obtained similar results for the long-term evolution of *q*, and for *p* in sparse networks without recombination, while we obtained even larger values of *p* at equilibrium for sparse networks with recombination (Figure S11), suggesting that connectivity density has a minor impact on the evolution of these parameters. These results are aligned with our previous study showing that network density and topology have only a small effect on the stability of networks of 10 genes [30].

Finally, the differences between eukaryotic and bacterial autoregulation values may also relate to the distinct regulatory processes of bacteria (e.g. common presence of operons) and eukaryotes (e.g. more widespread post-transcriptional regulation). As new circuits of transcription factor networks are elucidated in detail, the roles of negative and positive autoregulation in organismal development and evolution should be more clearly understood.

## Supporting Information

Figure S1 **Stability favors positive autoregulation.** Error bar chart of sign of autoregulation (*p*, Equation (6)) as a function of stability (Equation (3)) for two network sizes *N* = 4 and 10. This represents the inverse relationship of Figure 1B. Error bars represent standard deviations.
(EPS)

Figure S2 **Stability and cycles select for opposite signs of autoregulation.** Time evolution of *p* (Equation (6)) for different selection models. This reproduces Figure 2 with the added results of cycles of length up to 7, all in the same panel. Symbols and selection models are explained in Methods.
(EPS)

Figure S3 **Stability and cycles select for opposite signs of regulation.** Heat maps of the average matrix for different selection models. This is a different way of representing Figures 2 and S2, with the added information of the sign of off-diagonal elements. The matrices were averaged across individuals, populations and evolutionary time as explained in Results.
(EPS)

Figure S4 **Positive autoregulation favors robustness.** Same as Figures 6A and B but for random networks not pre-selected for stability. A. Boxplots of robustness (Equation (5)) for each *p* = 0, 0.1,…, 1. B. The inverse of (A), i.e., boxplots of *p* (Equation (6)) for 5 bins of increasing robustness, each of size 0.2, from 0 to 1.
(EPS)

Figure S5 **The sign of off-diagonal elements changes the relationship between robustness and autoregulation.** Robustness is not always maximized by *p* = 1 in stable networks. Here we show maximization at (*p* = 0.9, *q* = 0.65) and (*p* = 0.6, *q* = 0.70), for example. Networks are non-evolved. Lines serve as guides to the eye as *p* takes discrete values between 0 and 1.
(EPS)

Figure S6 **Engineering super-robust networks: there's more to the topology of robust networks.** Boxplots of robustness for specific values of *p* and *q*. A. Networks were engineered by combining the off-diagonal elements of stable networks with low *p* and a *q* similar to that of the equilibrium (*q* = 0.53 or 0.54), with a diagonal with high *p* (*p* = 0.9 or 1.0). B. Randomly sampled stable networks with same average *p* and *q* as the engineered ones.
(EPS)

Figure S7 **Robustness is not fully maximized in evolution.** Coevolution of *p* (A), *q* (B), stability (C), and robustness (D) under selection for stability (‘no-target’ model). Same as Figure 5 but for five different starting conditions: (*p*_0_, *q*_0_) = (0.5, 0.5), (0.5, 0.9), (0.5, 0.1), (0.9, 0.9), (0.9, 0.1). All networks in a population have the same (*p*_0_, *q*_0_) at the start of evolution.
(EPS)

Figure S8 **Positive long-range autoregulatory motifs are also associated with stability and robustness.** Same as Figure 5 with the inclusion of *r* given by Equation (7).
(EPS)

Figure S9 **Engineered networks have positive long-range autoregulatory interactions.** Boxplots of *r* (Equation (7)) for a subset of the networks shown in Figure S6. A. Stable networks with *p* = 0.1 and *q* = 0.54 (“engineered”). B. Stable networks with *p* = 0.9, *q* = 0.53 (“stable”). Population size is *n* = 10^4^ for each.
(EPS)

Figure S10 **Sign of regulation evolves to the same values with or without recombination.** Coevolution of *p* (A), *q* (B), stability (C), and robustness (D) with and without recombination. Black lines (recombination) are the same as Figure 5. Recombination is modeled as full chromosome segregation (no crossover) as described by Wagner [23].
(EPS)

Figure S11 **Autoregulation is even more positive for sparse networks with recombination.** Coevolution of *p* (A), *q* (B), stability (C), and robustness (D) for dense and sparse networks with and without recombination. Black lines (dense networks with recombination) are the same as Figure 5. Dense networks are fully connected (all genes regulate themselves and each other). Each gene in sparse networks regulates and is regulated by two other genes (average network degree of 2, as in biological networks [47]).
(EPS)
